# Impact of SGLT2 inhibitors on urinary tract infections in patients with diabetes mellitus and ileal urinary diversion: a retrospective cohort study

**DOI:** 10.1007/s11255-026-05175-4

**Published:** 2026-05-09

**Authors:** Nicolas Arnold, Ruby Hischier, Mihai Dorin Vartolomei, Laila Schneidewind, Nicola Giudici, Raphael Röthlisberger, Bernhard Kiss, Beat Roth

**Affiliations:** 1 Department of Urology, Inselspital, University Hospital of Bern, Wilhelm-Fabry-Haus, 3010 Bern, Switzerland; 2 The Institution Organizing University Doctoral Studies (I.O.S.U.D.), ‘Iuliu Hatieganu’ University of Medicine and Pharmacy, Cluj-Napoca-Napoca, Romania

**Keywords:** Sodium-Glucose Transporter 2 Inhibitors, Diabetes Mellitus, Urinary Tract Infection, Bacteriuria, Glucosuria, Cystectomy, Urinary Diversion

## Abstract

**Purpose:**

The impact of sodium–glucose linked transporter-2 inhibitors (SGLT2i) on urinary tract infection (UTI) risk remains uncertain and may be dose-dependent. No prior studies have evaluated their effect in patients with urinary diversion after cystectomy. This study evaluated the association between SGLT2i use and UTI in patients with diabetes mellitus (DM) after cystectomy and ileal urinary diversion.

**Methods:**

We conducted a retrospective single-center cohort study including all patients with DM who underwent cystectomy with ileal urinary diversion between March 1996 and November 2023. Primary outcome was UTI occurrence, assessed by incidence rate and infection-free survival (IFS). Secondary outcomes included asymptomatic bacteriuria and glucosuria. UTI incidence rate was compared using Poisson regression. IFS was evaluated using Kaplan–Meier curves, log-rank test and Cox regression analysis.

**Results:**

Among 123 included patients with DM and ileal urinary diversion, 18 (14.6%) received SGLT2i therapy. Median follow-up was 35 months (IQR 12–83). Overall, 121 UTI were documented, 6 (5.0%) in patients with and 115 (95.0%) without SGLT2i treatment, corresponding to incidence rates of 0.206 vs. 0.210 UTI/patient/year (*p* = 0.959). Median IFS was 9 months (IQR 3–31) in the SGLT2i and 17 months (IQR 5–74) in the non-SGLT2i group, with no significant difference (*p* = 0.489).

**Conclusion:**

In this cohort, SGLT2i therapy was not associated with an increased risk of UTI in patients with DM and ileal urinary diversion after cystectomy. These findings suggest no evident signal for increased infection risk; however, prospective studies are warranted to confirm these observations.

**Supplementary Information:**

The online version contains supplementary material available at https://doi.org/10.1007/s11255-026-05175-4.

## Introduction

Type 2 diabetes mellitus (DM) affects more than 360 million people worldwide and represents a global health challenge [1]. Sodium–glucose linked transporter 2 inhibitors (SGLT2i), developed for type 2 DM, have been widely used for over a decade [2, 3]. They are recommended as first- or second-line therapy, particularly in patients with additional cardiovascular disease, heart failure, or chronic kidney disease [4], and their efficacy has also been demonstrated in other forms of DM [5]. Beyond glucose lowering, SGLT2i reduce heart failure events and cardiovascular mortality and are now recommended even in non-diabetic patients with cardiovascular or renal disease [6]. Their mechanism involves inhibition of renal glucose reabsorption, resulting in glucosuria, improved HbA1c, weight loss, nephroprotection, and lower blood pressure [7]. Genital infections are well-recognized adverse effects, whereas the association between SGLT2i and urinary tract infections (UTI) remains controversial; some studies implicate glucosuria as a risk factor, but increased UTI rates were only reported with higher doses of dapagliflozin [8–10].

Type 2 DM itself predisposes to UTI, with worse prognosis and higher hospitalization rates [11, 12]. Patients with long-standing disease, poor glycemic control, or diabetic complications are particularly vulnerable [13]. Asymptomatic bacteriuria (ABU) is more common in patients with DM and represents a risk factor for UTI in this cohort, although treatment of ABU does not reduce complication rates [14, 15].

Bacteriuria is also highly prevalent in patients with urinary diversion, independent of the underlying indication [16]. While ABU is defined as ≥ 10^5^ cfu/mL in the general population, no uniform definition exists for patients with urinary diversion; some authors suggest ≥ 10^6^ cfu/mL [17, 18]. Reported risk factors include anatomical or functional abnormalities, residual urine, intermittent self-catheterization, and colonization at the mucocutaneous junction [19]. Despite frequent bacteriuria, patients often remain asymptomatic, likely due to distinct inflammatory responses of intestinal compared with bladder mucosa [17]. Although antibiotic prophylaxis may be used in recurrent UTI, it is not recommended for ABU [20]. Importantly, bacteriuria does not necessarily precede UTI, and the pathogenesis remains incompletely understood [21]. Nevertheless, UTI are among the most common complications and causes of readmission after cystectomy, particularly in patients with neobladder [22, 23]. DM further increases both perioperative and long-term risk of UTI [24, 25].

In this context, SGLT2i-induced glucosuria may represent an additional trigger for infection. Patients with urinary diversion already display high rates of bacteriuria, and the combination with enhanced glucosuria could further increase the risk of UTI. To date, no data are available on this potential interaction, which forms the rationale for the present study.

## Methods

### Study design

We retrospectively reviewed all cystectomy patients who underwent one of three types of ileal urinary diversion (ileal conduit, orthotopic ileal neobladder, catheterizable ileal pouch) at the Department of Urology, University Hospital Bern, between March 1996 and November 2023. Patients were eligible if they had been diagnosed with DM (type 1–3 [26]) before surgery or during postoperative follow-up. Individuals without follow-up at our institution were excluded. No formal sample size calculation was performed due to retrospective design. Ethical approval was obtained from the Institutional Review Board (IRB/KEK-BE 2016-00660). The study was conducted in accordance with the STROBE guidelines (Supplementary Table S1) and in accordance with the 1964 Declaration of Helsinki and its later amendments. Written informed consent was obtained from all participants.

### Outcome measurements

Primary outcome was the occurrence of UTI, assessed by incidence rate and infection-free survival (IFS). UTI was defined as the presence of symptoms related to the urinary diversion and/or systemic signs of inflammation (fever, flank pain, elevated inflammatory parameters, general deterioration in health, macrohematuria, other), based on the clinical assessment of the treating physician, and with or without a positive urine culture. Perioperative UTI within 30 days after cystectomy were excluded, as indwelling catheters and/or stents could have confounded microbial results. UTI episodes were identified during emergency consultations at our institution, or well described reports from general practitioners.

Secondary outcomes included ABU, defined as bacterial growth ≥ 10^3^ cfu/mL in routine urine culture without symptoms or inflammatory signs, and glucosuria, assessed as a direct correlate of SGLT2i therapy. Routine urine cultures were obtained in asymptomatic patients during regular follow-up at 3 and 6 months postoperatively, every 6 months for the first 5 years, and annually thereafter. Follow-up intervals could be modified for oncological or functional reasons. Glucosuria was determined using urine dipstick tests (five categories: negative, 3 mmol/L, 6 mmol/L, 17 mmol/L, 56 mmol/L) and was assessed during routine follow-up visits or emergency consultations at our institution.

Follow-up commenced 30 days after cystectomy if DM was already present before surgery, or on the day of DM diagnosis if patients developed DM during the postoperative period. For patients receiving SGLT2i, the follow-up period was limited to the duration of active therapy and was therefore stopped at treatment discontinuation. For patients initiating SGLT2i during follow-up, person-time was considered exposed only from the time of treatment initiation onward, while time prior to initiation was not included in the non-exposed group. Patients without UTI were censored for IFS at the time of their last follow-up visit. Loss to follow-up was handled by censoring patients at the date of their last follow-up visit, with time-to-event analyses assuming non-informative censoring.

### Statistical analysis

Categorical variables were reported as absolute numbers and percentages; continuous variables as medians with interquartile ranges (IQR). Fisher's exact and Mann–Whitney U test were used to compare categorical and continuous variables of patient groups, respectively. Incidence rate was compared using Poisson regression. IFS was analyzed using Kaplan–Meier curves and log-rank test. Univariate and multivariate Cox regression models were applied to evaluate associations between UTI and variables including age, sex, type of urinary diversion, type and onset of DM, SGLT2i therapy, UTI symptoms, hospitalization, urine culture results, and type and duration of antimicrobial therapy. Results were reported as hazard ratios (HR) with 95% confidence intervals (CI). A two-sided P value < 0.05 was considered statistically significant. A post hoc power analysis for the Cox model was performed using the observed hazard ratio and number of events. Sensitivity analyses were conducted using the proportional-hazards assumption. Missing data were not imputed due to the retrospective study design. Analyses were performed using available data (complete-case analysis). The proportion of missing data was low and assumed not to materially affect the results.

## Results

### General results

A total of 123 patients were included, comprising 89 men (72.4%) and 34 women (27.6%), with a median age of 70 years (IQR 62–77) and a median follow-up of 35 months (IQR 12–83). Ileal urinary diversion consisted of ileal conduit in 76 (61.8%), orthotopic ileal neobladder in 43 (35.0%), and catheterizable ileal pouch in 4 patients (3.2%). At time of cystectomy, 99 patients (80.5%) had pre-existing DM, whereas 24 (19.5%) developed DM during follow-up. Two patients (1.6%) had type 1, 112 (91.1%) type 2, and 9 (7.3%) type 3 DM. A total of 105 patients (85.4%) received either dietary or pharmacologic antidiabetic therapy other than SGLT2i, with a median follow-up of 50 months (IQR 40–88). Eighteen patients (14.6%) were treated with SGLT2i for a median of 11 months (IQR 3–31), all with type 2 DM. Empagliflozin was used in 10/18 (55.6%), dapagliflozin in 6/18 (33.3%), and canagliflozin in 2/18 patients (11.1%). Eight patients (44.4%) received continuous SGLT2i therapy throughout follow-up, while 10 (55.6%) initiated or discontinued therapy during the postoperative period. After treatment was discontinuation, none of the patients resumed SGLT2i therapy. Reasons for discontinuation included metabolic acidosis (*n* = 2), improved glycemic control after bariatric surgery (*n* = 1), and unknown cause (*n* = 1; Table 1).

**Table 1:** Patient characteristics

Variables	Total	SGLT2 +	SGLT2-	*p* value
Total patients, n (%)	123 (100)	18 (14.6)	105 (85.4)	
Follow-up, months				< 0.001
Median (IQR)	48 (14—88)	11 (3—31)	50 (14—88)	
Mean (SD)	62.2 (59.0)	19.4 (20.7)	62.6 (58.1)	
Sex, n (%)				0.151
Male	89 (72.4)	16 (88.9)	73 (69.5)	
Female	34 (27.6)	2 (11.1)	32 (30.5)	
Age, years				0.574
Median (IQR)	70 (62—77)	70 (62—75)	70 (62—77)	
Mean (SD)	68.4 (9.3)	68.6 (7.4)	68.4 (9.6)	
Type of ileal urinary diversion, n (%)				0.157
Ileum conduit	76 (61.8)	10 (55.6)	66 (62.9)	
Orthotopic ileal neobladder	43 (35.0)	6 (33.3)	37 (35.2)	
Catheterizable ileal pouch	4 (3.2)	2 (11.1)	2 (1.9)	
Type of DM, n (%)				0.529
1	2 (1.6)	0 (0.0)	2 (1.9)	
2	112 (91.1)	18 (100.0)	84 (89.5)	
3	9 (7.3)	0 (0.0)	9 (8.6)	
DM present at time of cystectomy, n (%)				1.000
Yes	99 (80.5)	15 (83.3)	84 (80.0)	
No	24 (19.5)	3 (16.7)	21 (20)	

*IQR* interquartile range, *SD* standard deviation, *DM* diabetes mellitus

Tests: Mann–Whitney U test, Fisher's exact test

### Primary outcome

**Fig. 1:**
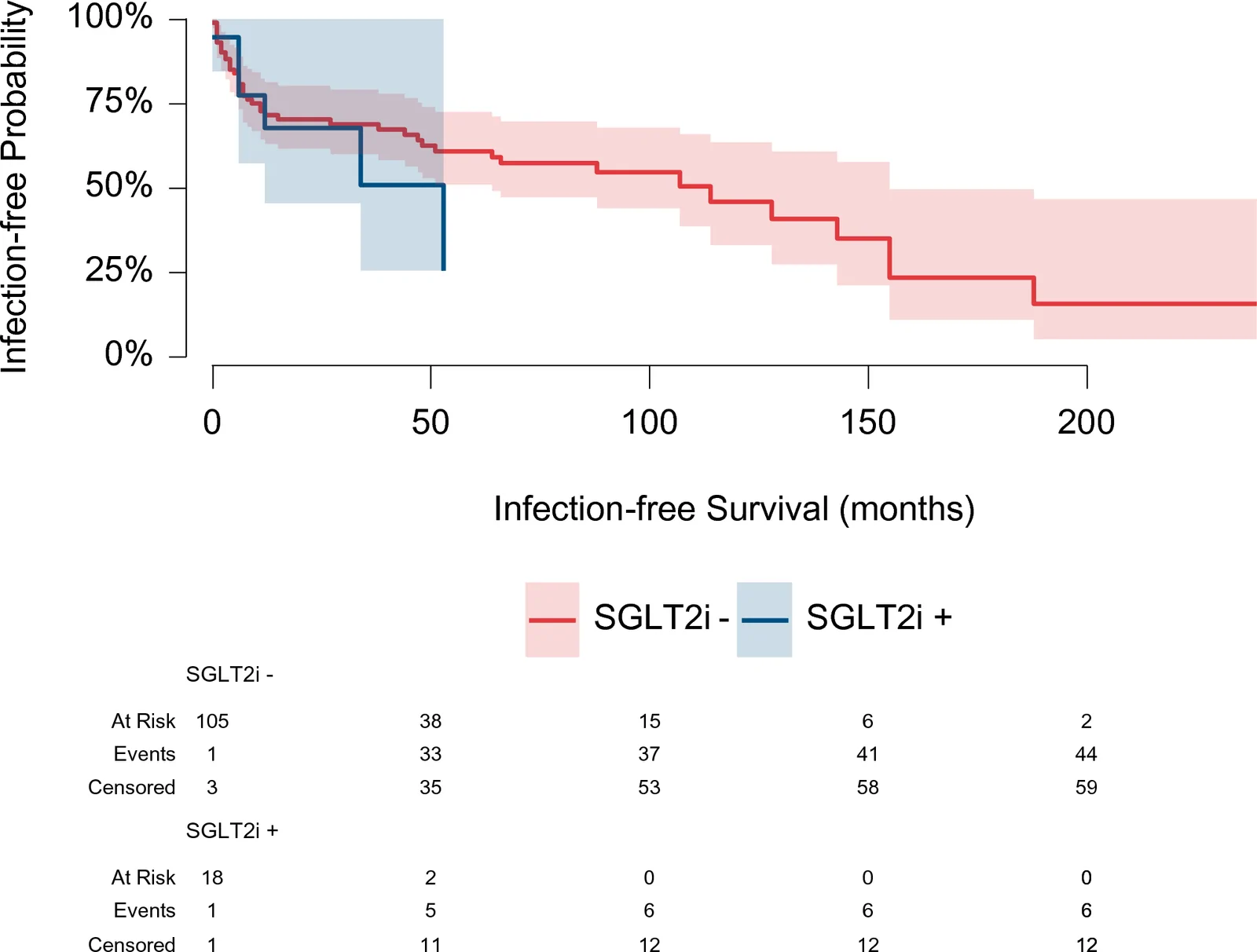
Kaplan–Meier curves illustrating infection-free survival in patients with (blue) and without (red) SGLT2i use

**Fig. 2:**
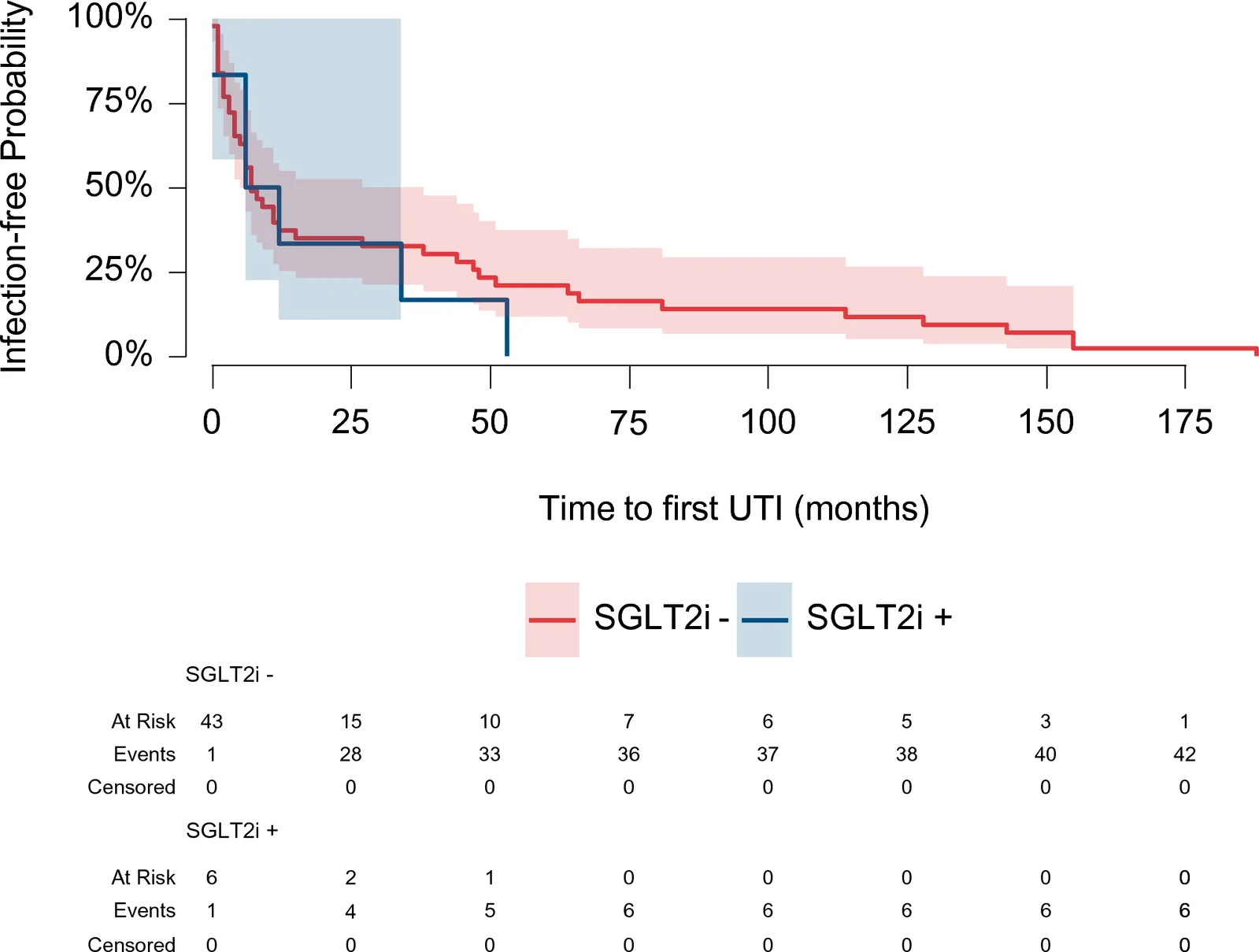
Kaplan–Meier curves illustrating time to first UTI in patients with (blue) and without (red) SGLT2i use

Overall, 121 UTI were documented: 6 UTI (5.0%) in 6/18 patients (33.3%) receiving SGLT2i and 115 UTI (95.0%) in 43/105 patients (41.0%) without SGLT2i, corresponding to an incidence rate of 0.206 vs. 0.210 UTIs per patient-year (*p* = 0.959), respectively. When stratified by type of urinary diversion, UTI incidence rates were 0.228 (0.314 vs. 0.224) for ileal conduit, 0.184 (0.187 vs. 0.101) for orthotopic neobladder, and 0.283 (0.152 vs. 0.358) UTI per patient-year for catheterizable pouch, respectively. 12/18 patients (66.6%) with and 62/115 (59.0%) patients without SGLT2i use did not experience UTI during follow-up. Median IFS was 9 months (IQR 3–31) versus 17 months (IQR 5–74), respectively (Fig. 1). When only considering patients who experienced UTI, median time to first UTI was 9 months (IQR 6–29) in the SGLT2i group and 7 months (IQR 3–48) in the non-SGLT2i group (Fig. 2). Kaplan–Meier and log-rank test showed no significant difference in IFS (*p* = 0.489) and time to first UTI (*p* = 0.571) between groups. Cox regression analysis did not identify variables significantly associated with IFS or time to first UTI (Supplementary Table S2 and S3). The proportional-hazards assumption was not violated, as the global test for the multivariable Cox model was non-significant (*p* = 0.054). Elevated inflammatory parameters were the most common clinical sign (89/126, 70.6%), and *Escherichia coli* was the predominant pathogen (41/126, 32.5%). Hospitalization was required in 65.9% (83/126) of UTI episodes. Ceftriaxone was the most frequently prescribed antibiotic (43/126, 34.1%), with a median treatment duration of 10 days (IQR 7–14; Table 2).

**Table 2:** Primary outcome

Variables	Total	SGLT2 +	SGLT2-	*p* value
Total UTI, n (%)	121 (100)	6 (5.0)	115 (95%)	
UTI				0.959
Incidence, UTI/patient/year	0.210	0.206	0.210	
IFS, months				0.489
Median (IQR)	16 (5—69)	9 (3—31)	17 (5—74)	
Mean (SD)	41.1 (49.8)	17.3 (17.5)	45.1 (52.3)	
Symptoms, n (%)				
Elevated inflammation parameters	89 (73.6)	1 (17.6)	88 (76,5)	
Fever	61 (50.4)	2 (33.3)	59 (51.3)	
General deterioration of health	33 (28.9)	2 (33.3)	31 (27.0)	
Flank pain	19 (15.7)	1 (17.6)	18 (15.7)	
Macrohematuria	5 (4.1)	1 (17.6)	4 (3.5)	
Other	9 (7.4)	1 (17.6)	8 (7.0)	
Detected pathogen in UTI, n (%)				
Escherichia coli	41 (33.9)	1 (17.6)	40 (34.8)	
Klebsiella pneumoniae	12 (9.9)	2 (33.3)	10 (8.7)	
Aerococcus urinae	8 (6.6)	0 (0.0)	8 (7.0)	
Pseudomonas aeruginosa	8 (6.6)	0 (0.0)	8 (7.0)	
Enterococcus faecalis	6 (5.0)	0 (0.0)	6 (5.2)	
Staphylococcus aureus	6 (5.0)	0 (0.0)	6 (5.2)	
Klebsiella oxytoca	6 (5.0)	1 (17.6)	5 (4.3)	
Antibiotic/antimycotic agent, n (%)				
Ceftriaxone	43 (35.5)	2 (33.3)	41 (35.7)	
Amoxicillin/Clavulanic acid	40 (33.1)	1 (17.6)	39 (33.9)	
Ciprofloxacin	27 (22.1)	0 (0.0)	27 (23.5)	
Trimethoprim/Sulfamethoxazole	14 (11.6)	1 (17.6)	13 (11.3)	
Fosfomycin	10 (8.3)	0 (0.0)	10 (8.7)	
Norfloxacin	10 (8.3)	0 (0.0)	10 (8.7)	
Duration of antibiotic therapy, days				0.117
Median (IQR)	10 (7—14)	7 (7—8)	10 (7—14)	
Mean (SD)	10.8 (5.0)	7.8 (1.5)	10.9 (5.1)	
Need for hospitalization, n (%)				0.645
Yes	79 (62.7)	3 (50)	76 (66.1)	
No	42 (33.3)	3 (50)	39 (33.9)	

*UTI* urinary tract infection, *IQR* interquartile range, *SD* standard deviation, *IFS* infection-free survival

Tests: Poisson regression, Log-rank test, Fisher's exact test, Mann–Whitney U test

### Secondary outcome

During routine follow-up, 534 urine cultures were obtained, of which 294 (55.1%) revealed ABU and 240 (44.9%) were negative or showed insignificant mixed flora. ABU was observed in 55.8% (29/52) of cultures in the SGLT2i group and in 55.0% (265/482) of cultures in the non-SGLT2i group (*p* = 0.902). *Escherichia coli* was the most frequent pathogen overall (120/294, 40.8%) and in the non-SGLT2i group (120/265, 45.3%), whereas *Staphylococcus aureus* predominated in the SGLT2i group (8/29, 27.6%). Antimicrobial treatment was administered in 89 of 294 ABU episodes (27.9%), including 7/29 (24.1%) and 82/265 (30.9%) cases in patients with and without SGLT2i therapy, respectively.

A total of 721 routine urine dipstick analyses were recorded, of which 165 (22.9%) indicated glucosuria. As expected, glucosuria was significantly more frequent in patients receiving SGLT2i (78/84, 92.9%) than in those without (87/637, 13.7%; *p* < 0.001). The median glucosuria level in the SGLT2i group was 56 mmol/L (Table 3).

**Table 3:** Secondary outcomes

Variables	Total	SGLT2 +	SGLT2-	*p* value
ABU, n (%)				0.902
Yes	294 (55.1)	29 (55.8)	265 (55.0)	
No (negative or mixed flora)	240 (44.9)	23 (44.2)	216 (45.0)	
Detected pathogen in ABU, n (%)				
Escherichia coli	120 (40.8)	6 (20.7)	114 (43.0)	
Klebsiella pneumoniae	35 (11.9)	4 (13.8)	31 (11.7)	
Klebsiella oxytoca	27 (9.2)	3 (10.3)	24 (9.1)	
Candida spp	24 (8.2)	8 (27.6)	16 (6.0)	
Staphylococcus aureus	20 (6.8)	2 (6.9)	18 (6.8)	
Pseudomonas aeruginosa	13 (4.4)	0 (0.0.)	13 (4.9)	
Proteus mirabilis	12 (4.1)	0 (0.0)	12 (4.5)	
Antibiotic/-mycotic treatment of ABU, n (%)				0.468
Yes	89 (30.3)	7 (24.1)	82 (30.9)	
No	205 (69.7)	22 (75.9)	183 (69.1)	
Glucosuria, n(%)				< 0.001
Yes	165 (22.9)	78 (92.9)	87 (13.7)	
No (negative)	556 (77.1)	6 (7.1)	550 (86.3)	
Glucosuria, n (%)				
Negative	556 (77.1)	6 (7.1)	550 (86.3)	
3 mmol/l	40 (5.6)	2 (2.4)	38 (6.0)	
6 mmol/l	29 (4.0)	12 (14.3)	17 (2.7)	
17 mmol/l	27 (3.7)	17 (20.2)	10 (1.6)	
56 mmol/l	69 (9.6)	47 (56.0)	22 (3.4)	

*ABU* asymptomatic bacteriuria

Tests: Fisher's exact test

## Discussion

This study is the first to investigate associations of SGLT2i therapy, which consistently induces glucosuria, and the occurrence of UTI in patients with ileal urinary diversion after cystectomy. While previous studies in the general populations have suggested a possible association between SGLT2i use, glucosuria, and UTI, our retrospective cohort did not demonstrate a statistically significant association.

The annual UTI incidence rate in our cohort was relatively low (0.2/patient-year), despite patients being at increased baseline risk. Comparisons with prior studies are difficult, as most have focused on perioperative or short-term outcomes. Thulin et al. reported UTI in 22.0% of patients based on postal questionnaires, with a risk ratio of 2.1 for DM patients, 45.0% of whom had experienced UTI in the preceding year [24]. Parker et al. analyzed 1248 cystectomy patients and observed 129 UTI, identifying DM as an independent risk factor with an odds ratio of 2.27 (*p* < 0.001), although their analysis was restricted to the first 90 days postoperatively [25]. Both studies underscore the impact of DM on UTI risk, but differences in study design, timing, and follow-up limit comparability with our findings.

Comparisons of ABU prevalence across studies are similarly limited by heterogeneity in definitions of clinically relevant bacteriuria. In a systematic review, Qu et al. reported bacteriuria rates ranging from 9.1% to 100.0% after cystectomy, reflecting variations in patient populations, definitions, and sampling time points [21]. In our cohort, ABU was detected in 55.1% of urine cultures, with no significant difference between patients with (55.8%) and without (55.0%) SGLT2i therapy (p = 0.902). These findings suggest that SGLT2i do not affect bacterial colonization in ileal urinary reservoirs. Whether this reflects a distinct inflammatory response of ileal mucosa [17] or simply the high baseline prevalence of bacteriuria in this population remains uncertain. The incidence of urinary tract infections caused by Candida spp. (27.6%) is surprisingly high, albeit not statistically significant, in patients receiving SGLT2i therapy. Given that an increased risk of genital mycotic infections has been demonstrated in patients taking SGLT2i, ABU/UTI caused by Candida spp. should be closely monitored in future studies[10].

As expected, glucosuria was significantly more common under SGLT2i therapy (92.9% vs 13.7%; *p* < 0.001), confirming good adherence. However, this did not translate into differences in UTI or ABU rates, contradicting the hypothesis that glucosuria itself constitutes a risk factor for UTI.

Our study has several limitations. Potential sources of bias include selection bias due to treatment allocation based on clinical judgment, and information bias from retrospective data analysis. Potential bias may also arise from variable timing of DM diagnosis and SGLT2i initiation relative to cystectomy, although SGLT2i start was not associated with IFS (HR 0.83, 95% CI 0.57–1.21, *p* = 0.330). In addition, substantial imbalances between groups including shorter median follow-up (11 vs. 50 months; *p* < 0.001), smaller sample size (*n* = 18 vs. 105), and fewer UTI events (6 vs. 115) in the SGLT2i group—limited statistical power (post hoc power ~ 27%) and represent an important limitation of this study. For patients receiving SGLT2i, follow-up was restricted to the duration of active therapy. Therefore, a formal time-dependent exposure modeling was not applied, and immortal-time bias cannot be fully excluded, reflecting a major limitation of our study. Nevertheless, our analysis did not reveal any expected bias toward a protective effect of SGLT2i. As there is no clear definition of UTI in patients with urinary diversion, our definition of UTI is based on the presence of various associated symptoms and, ultimately, the judgment of the treating physician, resulting in a rather imprecise definition and a potential risk of bias. Furthermore, data on glycemic control were unavailable, despite poor control being a recognized risk factor for UTI. Combined with the fact that existing data on HbA1c, total duration of DM , and diabetic complications are incomplete, this represents a significant confounding factor. [27, 28].

Despite these limitations, our findings provide valuable evidence in a clinically highly relevant area with no prior data, indicating that SGLT2i therapy does not promote occurrence of UTI or ABU in patients with ileal urinary diversion. Other strengths of our study include the real-world study setting, the long follow-up period, and the multidimensional assessment of outcomes. However, given the study limitations, our finding should be interpreted with caution, and patients with urinary diversion should be closely monitored when SGLT2i are prescribed. Larger prospective studies with standardized bacteriuria definitions and inclusion of glycemic parameters are warranted, particularly as SGLT2i indications continue to expand [29, 30].

## Conclusion

In this retrospective cohort, SGLT2i therapy was not associated with increased occurrence of UTI or ABU in diabetic patients after ileal urinary diversion. Given the limited sample size and number of events, these findings should be considered exploratory and interpreted with caution. While no clear signal for an increased infection risk was observed, the study does not allow definitive conclusions regarding the underlying mechanisms or clinical implications. Further prospective studies incorporating glycemic control and additional diabetes-related factors are required to better characterize the relationship between SGLT2i therapy and UTI risk in this population.

## Supplementary Information

Below is the link to the electronic supplementary material.

Supplementary file1 (DOCX 155 KB)

## Data Availability

The data that support the findings of this study are available from the corresponding author upon reasonable request. The data are not publicly available due to privacy or ethical restrictions.

## Abbreviations

DM: Diabetes mellitus
SGLT2i: Sodium–glucose linked transporter 2 inhibitor
UTI: Urinary tract infections
ABU: Asymptomatic bacteriuria
IFS: Infection-free survival
