# Supplementary material for: Impact of SGLT2 inhibitors on urinary tract infections in patients with diabetes mellitus and ileal urinary diversion: a retrospective cohort study
Source: Int Urol Nephrol. 2026 May 9;58(10):4169–77. doi: 10.1007/s11255-026-05175-4 (PMC13658336; doi:10.1007/s11255-026-05175-4)
Supplement: Supplementary file 1 [file 11255_2026_5175_MOESM1_ESM.docx]

**Supplementary Table S1.** STROBE Statement

|  | | Item No | Recommendation | | Page |  |  |
| --- | --- | --- | --- | --- | --- | --- | --- |
| **Title and abstract** | | 1 | (*a*) Indicate the study’s design with a commonly used term in the title or the abstract | | Title,  Abstract |  |  |
|  |  |  | (*b*) Provide in the abstract an informative and balanced summary of what was done and what was found | | Abstract |  |  |
| Introduction | | | | |  |  |  |
| Background/rationale | | 2 | Explain the scientific background and rationale for the investigation being reported | | 3-4 |  |  |
| Objectives | | 3 | State specific objectives, including any prespecified hypotheses | | 3-4 |  |  |
| Methods | | | | |  |  |  |
| Study design | | 4 | Present key elements of study design early in the paper | | 4 |  |  |
| Setting | | 5 | Describe the setting, locations, and relevant dates, including periods of recruitment, exposure, follow-up, and data collection | | 4-5 |  |  |
| Participants | | 6 | (*a*) *Cohort study*—Give the eligibility criteria, and the sources and methods of selection of participants. Describe methods of follow-up | | 4-5 |  |  |
|  |  |  | (*b*) *Cohort study*—For matched studies, give matching criteria and number of exposed and unexposed | | N/A |  |  |
| Variables | | 7 | Clearly define all outcomes, exposures, predictors, potential confounders, and effect modifiers. Give diagnostic criteria, if applicable | | 5 |  |  |
| Data sources/ measurement | | 8 | For each variable of interest, give sources of data and details of methods of assessment (measurement). Describe comparability of assessment methods if there is more than one group | | 5 |  |  |
| Bias | | 9 | Describe any efforts to address potential sources of bias | | 6 |  |  |
| Study size | | 10 | Explain how the study size was arrived at | | N/A |  |  |
| Quantitative variables | | 11 | Explain how quantitative variables were handled in the analyses. If applicable, describe which groupings were chosen and why | | 6 |  |  |
| Statistical methods | | 12 | (*a*) Describe all statistical methods, including those used to control for confounding | | 6 |  |  |
|  |  |  | (*b*) Describe any methods used to examine subgroups and interactions | | 6 |  |  |
|  |  |  | (*c*) Explain how missing data were addressed | | 6 |  |  |
|  |  |  | (*d*) *Cohort study*—If applicable, explain how loss to follow-up was addressed | | 5-6 |  |  |
|  |  |  | (*e*) Describe any sensitivity analyses | | 6 |  |  |
| Results | | | |  | | | |
| Participants | 13 | (a) Report numbers of individuals at each stage of study—eg numbers potentially eligible, examined for eligibility, confirmed eligible, included in the study, completing follow-up, and analysed | | N/A \| \| | | | |
|  |  | (b) Give reasons for non-participation at each stage | | N/A \| \| | | | |
|  |  | (c) Consider use of a flow diagram | | N/A \| \| | | | |
| Descriptive data | 14 | (a) Give characteristics of study participants (eg demographic, clinical, social) and information on exposures and potential confounders | | 6-7 \| \| | | | |
|  |  | (b) Indicate number of participants with missing data for each variable of interest | | 6 \| \| | | | |
|  |  | (c) *Cohort study*—Summarise follow-up time (eg, average and total amount) | | 6-7 \| \| | | | |
| Outcome data | 15 | *Cohort study*—Report numbers of outcome events or summary measures over time | | 7-8 \| \| | | | |
| Main results | 16 | (*a*) Give unadjusted estimates and, if applicable, confounder-adjusted estimates and their precision (eg, 95% confidence interval). Make clear which confounders were adjusted for and why they were included | | Tables \| \| | | | |
|  |  | (*b*) Report category boundaries when continuous variables were categorized | | N/A \| \| | | | |
|  |  | (*c*) If relevant, consider translating estimates of relative risk into absolute risk for a meaningful time period | | N/A \| \| | | | |
| Other analyses | 17 | Report other analyses done—eg analyses of subgroups and interactions, and sensitivity analyses | | 7-8 \| \| | | | |
| Discussion | | | |  | | | |
| Key results | 18 | Summarise key results with reference to study objectives | | 8-10 \| \| | | | |
| Limitations | 19 | Discuss limitations of the study, taking into account sources of potential bias or imprecision. Discuss both direction and magnitude of any potential bias | | 10 \| \| | | | |
| Interpretation | 20 | Give a cautious overall interpretation of results considering objectives, limitations, multiplicity of analyses, results from similar studies, and other relevant evidence | | 8-10 \| \| | | | |
| Generalisability | 21 | Discuss the generalisability (external validity) of the study results | | N/A \| \| | | | |
| Other information | | | |  | | | |
| Funding | 22 | Give the source of funding and the role of the funders for the present study and, if applicable, for the original study on which the present article is based | | 16 \| \| | | | |

| **Supplementary Table S2. Univariate and multivariate Cox regression analysis for IFS** | | | | | |  |  |
| --- | --- | --- | --- | --- | --- | --- | --- |
|  | **Univariate** | | |  | **Multivariate** | | |
| **Variables** | **HR** | **CI 95%** | **p value** |  | **HR** | **CI 95%** | **p value** |
| Sex | 1.08 | 0.58-2.01 | 0.804 |  | 0.59 | 0.24-1.44 | 0.246 |
| Age | 1.00 | 0.97-1.03 | 0.731 |  | 0.98 | 0.95-1.02 | 0.286 |
| Type of UD | 0.85 | 0.50-1.47 | 0.578 |  | 0.49 | 0.22-1.10 | 0.083 |
| Type of DM | 0.69 | 0.24-1.99 | 0.496 |  | 1.15 | 0.15-8.96 | 0.895 |
| SGLT2i use (yes) | 1.34 | 0.56-3.22 | 0.571 |  | 1.82 | 0.61-5.47 | 0.286 |
| IFS = infections-free survival; UD = urinary diversion; DM = diabetes mellitus; SGLT2i = sodium-glucose linked transporter 2 inhibitor | | | | | | | |

| **Supplementary Table S3. Univariate and multivariate Cox regression analysis for time to first UTI** | | | | | | |  |
| --- | --- | --- | --- | --- | --- | --- | --- |
|  | **Univariate** | | |  | **Multivariate** | | |
| **Variables** | **HR** | **CI 95%** | **p value** |  | **HR** | **CI 95%** | **p value** |
| Sex | 0.88 | 0.47-1.64 | 0.684 |  | 0.80 | 0.32-2.00 | 0.682 |
| Age | 1.02 | 0.99-1.05 | 0.332 |  | 1.01 | 0.98-1.05 | 0.396 |
| Type of UD | 0.71 | 0.43-1.18 | 0.181 |  | 0.64 | 0.29-1.43 | 0.274 |
| Type of DM | 0.64 | 0.21-1.90 | 0.418 |  | 1.05 | 1.13-8.35 | 0.963 |
| SGLT2i use (yes) | 1.28 | 0.54-3.07 | 0.573 |  | 1.69 | 0.54-5.22 | 0.366 |
| UTI = urinary tract infection; UD = urinary diversion; DM = diabetes mellitus; SGLT2i = sodium-glucose linked transporter 2 inhibitor | | | | | | | |
